# Editorial: Emerging Advances in Bio-Nano Engineered Approaches Toward Intelligent Nanomedicine

**DOI:** 10.3389/fbioe.2021.703227

**Published:** 2021-05-24

**Authors:** Junqing Wang, Lin Mei, Wei Tao, Gang Liu

**Affiliations:** ^1^School of Pharmaceutical Sciences (Shenzhen), Sun Yat-sen University, Shenzhen, China; ^2^Tianjin Key Laboratory of Biomedical Materials, Key Laboratory of Biomaterials and Nanotechnology for Cancer Immunotherapy, Institute of Biomedical Engineering, Chinese Academy of Medical Sciences, Peking Union Medical College, Tianjin, China; ^3^Center for Nanomedicine, Brigham and Women's Hospital and Harvard Medical School, Boston, MA, United States; ^4^State Key Laboratory of Molecular Vaccinology and Molecular Diagnostics, Center for Molecular Imaging and Translational Medicine, School of Public Health, Xiamen University, Xiamen, China

**Keywords:** drug delivery, nanomedicine, bio-nano, nanovaccines, auto-PDT, SPIONs, exosomes, nanoMOFs

In the past two decades, nanomedicine has made tremendous progress in the fields of diagnosis, treatment, and prevention of diseases. With the continuous deepening of the knowledge in nanobiology, nanomedicine has rapidly evolved from the early stage of classic functional nanoparticles (e.g., liposomes, iron oxide nanoparticles, semiconductor/graphene quantum dots, colloidal gold nanoparticles, etc.) to a variety of bio-nano fusion systems with the “intelligent” use of nano-biological effects. This paradigm shift will bring about fundamental changes in drug delivery strategies (Zhang et al., 2018), companion diagnostics (Cai et al., 2018; Liu et al., 2021), synchronous therapy (Shi et al., 2020), as well as in the way of manipulating bio-nano interactions for medical applications (Tao et al., 2019). In this Research Topic, we present six representative original articles highlighting the recent research advances in design and concepts of novel bio-nano engineered approaches that cover multiple applications, including polymer-lipid hybrid nanovaccine for anti-tumor therapy, differently charged super-paramagnetic iron oxide nanoparticles (SPIONs) for M2 to M1 macrophages polarization, immuno-inert nanoMOFs for cancer therapy, self-driven photodynamic therapy (PDT), exosomes-based osteoporosis therapy, and nanomedicine for chronic pain relief.

Wang et al. described an effective prophylactic anti-cancer nanovaccine approached by co-encapsulation of lipidated TLR7/8 agonists and ovalbumin-derived peptide (SIINFEKL) in core-shell polymer–lipid hybrid nanoparticles (PLNs). This research attempts to address the major limitations of conventional vaccine formulation, including the poor tolerability profile of adjuvants, instability of peptide antigens, insufficient cellular uptake, rapid diffusion from post-injection, and systemic adverse effects. They have demonstrated that lipidation of adjuvant and peptide epitope following PLN encapsulation can largely improve anti-tumor immune responses and achieve desired *in vivo* prophylactic efficacy. In comparison to aluminum-contained vaccine formulation, PLN vaccine exhibited minimally systemic exposure, enhanced encapsulation efficacy, persistent antigen-presenting cellular uptake, and prolonged immune surveillance effect. The integration of lipidation modification and the PLN approach developed by the authors could be an important concept to direct the future success of anti-cancer nanovaccines.

Understanding the interactions between biological systems and nanoscale entities play a crucial role in the development of the biomedical application of nanomedicine (Liu et al., 2021). In this research topic, Zhang et al. presented a hypothesis that differently charged SPIONs would exhibit variable abilities to induce the conversion of the macrophage phenotype. The authors designed three different charged SPIONs (i.e., S+ positive, SN neutral, and S– negative) and investigated their intrinsic re-polarization effect on tumor-associated macrophages (TAMs), as well as their influence on tumor growth rate. They found that both S+ and S– could effectively reprogram TAMs from an M2 to an M1 phenotype at cellular level. This is possibly due to their high cellular uptake. To further study the *in vivo* antitumor activity, the authors co-injected the TAMs (pre-treated with SPIONs) with the tumor cells to establish a tumor xenograft. The tumor growth was significantly attenuated by S+ and S–, but S– exhibited enhanced cytotoxicity at higher concentrations. They concluded that S+ may be considered for further clinical anti-tumoral evaluation.

In another original work, Li et al. designed a “stealth” nanoMOFs for safe and efficient anti-cancer therapy. They engineered the MIL-100(Fe) nanoMOF via a step approach. Initially, the drug molecules (Gem-MP) were loaded into the pores and on the surfaces of the nanoMOF through the impregnation in aqueous solutions; then, the Gem-MP-loaded nanoMOF was further modified by the surface coating of DOPC and DSPE-PEG 2000 in alcoholic solution. This work showed that the PEGylation of drug-loaded MIL-100(Fe) can not only influence the drug release rate but also regulate the degradation process. The authors showed that PEGylation of NanoMOFs can significantly reduce the human serum albumin adsorption, macrophage uptake, and exhibited notable *in vitro* anticancer activity. However, a further *in vivo* study is encouraged to explore their therapeutic potential.

In a review article, Xie et al. highlighted the important role of exosomes in the pathogenesis of age-related osteoporosis. They initially introduced the basic characteristics of exosomes, followed by the discussion of regulatory pathways in mesenchymal stem cells (MSCs) differentiation mediated by exosomes. Then, they summarized the recent findings regarding osteoblast proliferation and osteoclastogenesis effects stimulated by exosomes. They also pointed out the negative regulatory effects of exosomes on osteoclast differentiation. In the end, the authors commented on the opportunities and challenges of clinical translation on utilizing exosomes for treating osteoporosis. In another mini-review, Chen et al. briefly introduced the latest nanotechnological approaches for managing chronic pain. The application of Liposomes, PLGA nanoparticles, carbon-based, and other inorganic nanomaterials for the targeted/non-targeted neuropathic pain relief were highlighted. Moreover, the authors provided a detailed discussion in considerations of nanomaterial design for chronic pain relief and exemplified future strategies that can be explored, such as oral, inhalation, sprayable nanomaterials, electrical stimulation, microneedles, targeted delivery, etc. In another mini-review, Blum et al. introduced the concept of self-exciting photodynamic therapy (auto-PDT) to overcome the major limitation of conventional PDT, which is the insufficient tissue penetration depth of light. The auto-PDT nanoplatforms can drive the PDT process without the presence of an external light source. However, these platforms are usually designed in response to oxidative chemical excitation in the manner of chemiluminescence or Cherenkov luminescence (radiological excitation). The authors critically reviewed the design strategies, mechanisms of excitation, and the therapeutic effect of auto-PDT nanoplatforms with state-of-the-art researches. In addition, the authors also provided a detailed discussion of the recent studies of auto-PDT nanotherapeutics concerning design strategy, challenges, and themes in their synthesis and application.

Overall, this collection of articles contributes to the research topic with innovative designs and concepts in intelligent nanomedicine. The bio-nano engineered approach is not only the key strategy to overcome the intrinsic limitations of nanomaterials but also opening doors to new opportunities for patients. The fast pace and emerging breakthroughs in bio-nanotechnology will motivate and inspire both research and industry to accelerate these interdisciplinary translations to clinical applications.

## Author Contributions

All authors listed have made a substantial, direct and intellectual contribution to the work, and approved it for publication.

## Conflict of Interest

The authors declare that the research was conducted in the absence of any commercial or financial relationships that could be construed as a potential conflict of interest.

## References

[B1] Cai W. Wang J. Liu H. Chen W. Wang J. Du L.. (2018). Gold nanorods@metal-organic framework core-shell nanostructure as contrast agent for photoacoustic imaging and its biocompatibility. J. Alloys Compds 748, 193–198. 10.1016/j.jallcom.2018.03.133

[B2] Liu Y. Wang J. Xiong Q. Hornburg D. Tao W. Farokhzad O.C. (2021). Nano–bio interactions in cancer: from therapeutics delivery to early detection. Acc. Chem. Res. 54, 291–301. 10.1021/acs.accounts.0c00413 33180454

[B3] Shi Y. Wang J. Liu J. Lin G. Xie F. Pang X.. (2020). Oxidative stress-driven DR5 upregulation restores TRAIL/Apo2L sensitivity induced by iron oxide nanoparticles in colorectal cancer. Biomaterials 233:119753. 10.1016/j.biomaterials.2019.119753 31923762

[B4] Tao W. Wang J. Parak W.J. Farokhzad O.C. Shi J. (2019). Nanobuffering of pH-responsive polymers: a known but sometimes overlooked phenomenon and its biological applications. ACS Nano 13, 4876–4882. 10.1021/acsnano.9b01696 30985108 PMC6748625

[B5] Zhang P. Wang J. Chen H. Zhao L. Chen B. Chu C.. (2018). Tumor microenvironment-responsive ultrasmall nanodrug generators with enhanced tumor delivery and penetration. J. Am. Chem. Soc. 140, 14980–14989. 10.1021/jacs.8b09396 30359020

